# Long-term effects of maternal exposure to Di (2-ethylhexyl) Phthalate on sperm and testicular parameters in Wistar rats offspring

**Published:** 2012-01

**Authors:** Mehran Dorostghoal, Ahmad Ali Moazedi, Adel Zardkaf

**Affiliations:** Department of Biology, Faculty of Sciences, Shahid Chamran University of Ahwaz, Ahwaz, Iran.

**Keywords:** *Di (2-ethylhexyl) Phthalate*, *Testis*, *Sperm*, *Morphometry*, *Stereology*

## Abstract

**Background:** Phthalate esters have been shown to cause reproductive toxicity in both developing and adult animals.

**Objective:** This study was designed to assess long-term effects of maternal exposure to Di (2-ethylhexyl) Phthalate (DEHP) on reproductive ability of both neonatal and adult male offspring.

**Materials and Methods:** 60 female rats randomly divided in four equal groups; vehicle control and three treatment groups that received 10, 100 and 500 mg/kg/day DEHP via gavage during gestation and lactation. At different ages after birth, the volumes of testes were measured by Cavellieri method, testes weights recorded and epididymal sperm samples were assessed for number and gross morphology of spermatozoa. Following tissue processing, seminiferous tubules diameter and germinal epithelium height evaluated with morphometric techniques.

**Results:** Mean testis weight decreased significantly (p<0.05) in 500 mg/kg/day dose group from 28 to 150 days after birth. Significant decreases were seen in total volumes of testis in 100 (p<0.05) and 500 (p<0.01) mg/kg/day doses groups until 150 days after birth. Seminiferous tubules diameter and germinal epithelium height decreased significantly in 100 (p<0.05) and 500 (p<0.01) mg/kg/day doses groups during postnatal development. Also, mean sperm density in 100 mg/kg/day (p<0.05) and 500 mg/kg/day (p<0.01) doses groups and percent of morphologically normal sperm in highest dose group (p<0.05) decreased significantly until 150 days after birth.

**Conclusion:** Present study showed that maternal exposure to Di (2-ethylhexyl) Phthalate during gestation and lactation caused to permanent and dose-related reductions of sperm and testicular parameters in rats offspring.

## Introduction

In recent years, there has been an increasing concern about the potential adverse effects of occupational and environmental exposures to endocrine disrupting chemicals (EDCS) in affecting human and wildlife health (1, 2). This caused declining sperm concentration and male infertility (3, 4). EDCS consist of synthetic and naturally occurring chemicals that affect the balance of normal hormonal functions in animals (5). These compounds mimic the sex hormones, estrogen or androgen, or block the activities of estrogen or androgen, i.e., are anti-estrogens or anti-androgens (6). 

Phthalates esters are one of the most important classes of endocrine disrupting chemicals that constitute 10-60% by weight of many polyvinyl chloride plastics. These chemical compounds are responsible of flexibility, transparency and other desirable physical properties of plastics (7). Because Phthalate esters are not covalently bound to the polymers, they can leach into the foods, beverages, or other materials and have been detected in several human bodily fluids, including amniotic fluid (8), breast milk (9) and maternal urine during pregnancy (10). 

Di (2-ethylhexyl) Phthalate (DEHP), the most abundantly Phthalates found in the environment (11), was used as a plasticizer in products such as food packaging, children’s products (toys and crib bumpers) and medical equipments (12). Epidemiological studies revealed that there are correlations between urinary concentrations of metabolites of Phthalates and incidence of anomalies such as cryptorchidism and shortened anogenital distance of newborn males (10, 13). 

Also, several developmental studies have been shown that DEHP can affect spermatogenesis in adults and impair development of fetal male rats caused to deformities such as undescended testes, hypospadias, epididymal agenesis and a reduction of the anogenital distance (14-17). Degeneration of seminiferous epithelium and decreased testis weight were seen in male offspring rats exposed maternally during gestation to high dose (750 mg/kg/day) of DEHP (14). 

Testis weight and epididymal sperm counts reduced in rat’s exposure to DEHP during lactation (14, 18, 19). Associations between Phthalate exposure and shortened anogenital index have been reported in infant boys whose mothers had elevated urine levels of Phthalate metabolites during pregnancy (10). 

Exposure to DEHP during gestation and lactation affect sexual development and reduces testis weight and alters testicular morphology in rats (20). In utero and lactational exposures to 10 or 750 mg/kg DEHP reduced significantly serum testosterone levels in male rats offspring at 21 days of age (21). 

However, although it is well known that Phthalate esters induce testicular dysfunction in both adult and immature rats, little is still known regarding the long-term effects of DEHP on the testicular function of male rats that maternally exposed to it during gestation and lactation periods. So, present study was undertaken to investigate long-term morphometric and stereologic effects of in utero and lactational exposure to different doses of Di (2-ethylhexyl) Phthalate on semen and testicular parameters in Wistar rats’ offspring during neonatal, prepubertal and postpubertal periods.

## Materials and methods

**Animals**

In this experimental study, Wistar rats were obtained from animal house of Jundishapour Medical Sciences University of Ahwaz and kept under specific conditions on a constant 12-hours light/dark cycle and at a controlled temperature of 22±2°C. Standard pellet food and distilled water were available ad libitum. 60 Female Wistar rats (100±10 days old) were mated overnight at the proportion of three females per male. Vaginal smears were collected daily and examined for the presence of sperm. The day of sperm detection in vaginal smears was considered day 0 of pregnancy. 

Female rats randomly divided in four equal groups (220±10 g and n=15 per group); vehicle control and three treatment groups that received 10, 100 and 500 mg/kg/day Di (2-ethylhexyl) Phthalate (Merck Co., Germany) via gavage during gestation and lactation. The doses were established from related studies of reproductive toxicity (14, 22, 23). DEHP was dissolved daily in 10 ml/ kg of body weight corn oil (Ghoncheh Co., Iran) as vehicle before administration (22). 

The animals were treated daily by oral gavages (14, 22, 23) from day 3 of pregnancy to day 21 of lactation. At 1, 7, 21, 28, 60, 90, 120 and 150 days of age 5 pups were randomly selected (24), weighed and under chloroform inhalation anesthesia, their testes were removed. At 7 and 14 days of age testes were removed, weighed and fixed by immersion in Bouin’s solution for 24 hours. At 21, 28, 60, 90, 120 and 150 days of age right testes were removed and weighed, and left testes fixed by whole body perfusion with Bouin’s solution, then removed and post-fixed by immersion in the same solution for 24 hours.

**Morphometrical analysis**

Tissue samples from right testes were excised, and processed for paraffin embedding sections. Serial sections with 5μm thickness were stained with heamatoxylin and eosin and used for morpho metrical studies at light microscopic level. For measuring of seminiferous tubule diameter and germinal epithelium height, 90 round or nearly round cross-sections of seminiferous tubules were randomly chosen in each rat. 

Then, using an ocular micrometer of light microscopy (Olympus BH), at a magnification of ×40, two perpendicular diameters of each cross- section of seminiferous tubules were measured and the mean of these was calculated. Also, germinal epithelium height in 4 equidistance of each cross-section of seminiferous tubules measured and the mean of these was calculated (25).

**Stereological analysis**

In every sampling stage the volumes of left testes were measured by Cavellieri method (26). For this purpose, each testis was embedded in paraffin and serial 5 µm sections, with a random start, were prepared along the long axis of the organ, then those were stained with haematoxylin- eosin and were used for quantitative analysis. 

Then, 10 Cavallieri sections were taken at the length of each testis. A test grid with 100 points super imposed on the photograph of each section and number of points overlying it was counted. Then, absolute volume of each test is calculated by Cavellieri equation (26).

**Sperm analysis**

Epididymis was separated carefully from the right testis. Semen samples of epididymal tail were assessed for number and gross morphology of sperm without the investigator knowing which samples were from which group. The sperm heads were counted at 200×magnification and expressed as million/ml of suspension (27). 

The sperm morphology was also determined using Eosin-Nigrosin staining method (28). Two hundred sperm per animal were examined microscopically at 40-100×magnifications, and the number of morphologically abnormal sperm was recorded to give the percentage of abnormal sperm.

**Statistical analysis**

All data were analyzed using SPSS version 10.0 for windows. Semen and testicular parameters in different groups were compared by one-way ANOVA and Tukey’s test was used as a Post hoc test. Differences were considered to be significant when p<0.05.

## Results

**Figure 1:**
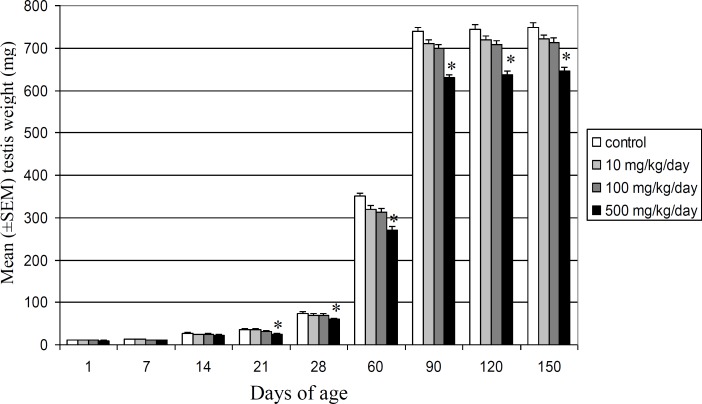
Comparison of mean (±SEM) testis weights (mg) in control and DEHP-treated Wistar rats’ offspring during different stages of postnatal development (*p<0.05).

Mean testis weight showed significant (p<0.05) decreases in highest dose group from 21 to 150 days of postnatal development, whereas no statistically significant differences were observed in 10 and 100 mg/kg/day DEHP doses groups in comparison with control vehicle group (Figure 1). Testis weight of offspring in highest dose group decreased significantly for the first time at 21 days after birth and reduced 22.56% and 13.82% at 60 and 150 days of age, respectively. 

**Figure 2:**
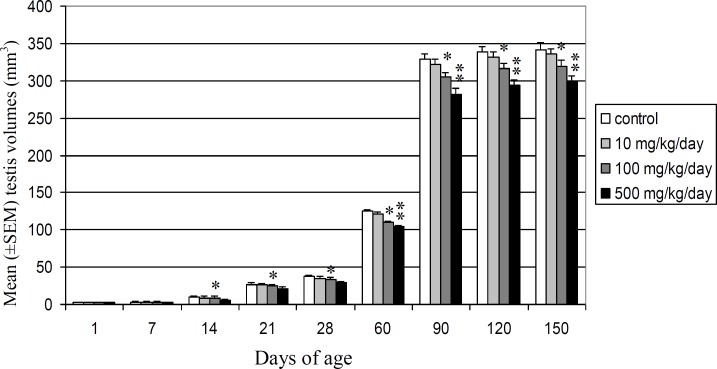
Comparison of mean (±SEM) testis volumes (mm^3^) in control and DEHP-treated Wistar rats’ offspring during different stages of postnatal development (*p<0.05, **p<0.01).

Mean absolute volume of testis decreased significantly (p<0.05) in 100 mg/kg/day DEHP dose group from 60 to 150 days after birth. It also showed significant reduction in highest dose group at 14, 21 and 28 days (p<0.05) and at 60, 90 and 150 (p<0.01) days after birth, but no statistically significant differences were observed in 10 mg/kg/day dose group in comparison with control vehicle group (Figure 2). Testis volume of Wistar rats offspring decreased significantly at 14 days after birth for the first time and reduced 17.18% and 12.70% at 60 and 150 days of age, respectively, in highest dose group. Testis volume of offspring reduced 12.50% and 6.71% in 100 mg/kg/day DEHP dose group at 60 and 150 days of age, respectively. Mean seminiferous tubules diameter decreased significantly (p<0.05) in 100 mg/kg/day DEHP dose group from 21 to 150 days after birth. Also, significant (p<0.01) decrease was seen in highest dose group from 7 to 150 days after birth in comparison with control vehicle group. 

**Figure 3:**
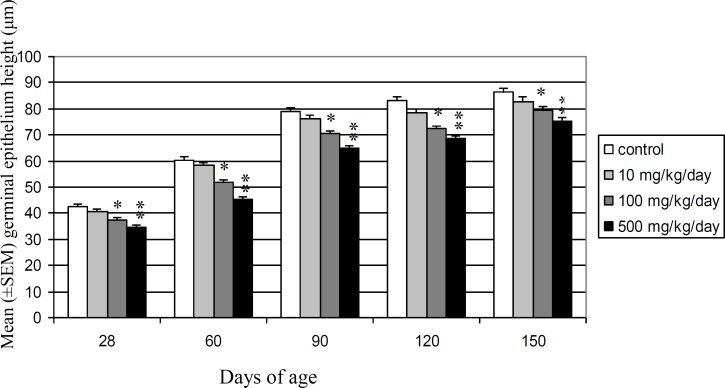
Comparison of mean (±SEM) germinal epithelium height (μm) in control and DEHP-treated Wistar rats’ offspring during different stages of postnatal development (*p<0.05, **p<0.01).

There were no significant differences between 10 mg/kg/day DEHP dose groups and control vehicle groups (Table I). Seminiferous tubules diameter in 100 and 500 mg/kg/day DEHP doses groups decreased significantly for the first time at 21 and 1 days after birth, respectively. Seminiferous tubule diameter at 60 and 150 days of age reduced 13.11% and 7.23% in 100 mg/kg/day DEHP dose group and 18.44% and 12.13% in 500 mg/kg/day DEHP dose group, respectively. Mean germinal epithelium height showed significant decrease from 28 to 150 days after birth in 100 (p<0.05) and 500 (p<0.01) mg/kg/day DEHP doses groups, but no statistically significant differences were observed in 10 mg/ kg/ day DEHP dose group in comparison with control vehicle group (Figure 3). Germinal epithelium height reduced 11.57% and 8.17% in 100 mg/kg/day DEHP dose group and 18.40% and 12.64% in 500 mg/kg/day DEHP dose group at 60 and 150 days of age, respectively. 

Significant decrease observed in mean sperm density from 60 to 150 days after birth in 100 (p<0.05) and 500 (p<0.01) mg/kg/day DEHP doses groups in comparison with control group (Table II). Sperm production reduced 14.50% and 11.90% in 100 mg/kg/day DEHP dose group and 26.65% and 23.59% in 500 mg/kg/day DEHP dose group at 60 and 150 days of age, respectively. Also, mean percent of morphologically normal sperm decreased significantly (p<0.05) in 500 mg/kg/day DEHP dose group from 60 to 150 days after birth in comparison with control vehicle group (Table II). 

**Table I:** Comparison of mean (±SEM) seminiferous tubules diameter (μm) in control and DEHP-treated Wistar rats offspring during different stages of postnatal development

**Groups**	**Days after birth**								
	**1**	**7**	**14**	**21**	**28**	**60**	**90**	**120**	**150**
Control	48.22±1.7	59.82±1.6	75.20±0.5	86.56±2.3	123.00±3.4	182.30±9.2	259.83±20.7	261.83±18.6	264.44±21.3
10 mg/kg/day	46.33±1.5	59.60±1.8	72.13±1.2	84.77±3.1	120.32±4.1	179.75±7.3	253.88±17.6	255.88±18.1	257.76±17.4
100 mg/kg/day	45.18±1.4	59.31±1.3	69.69±0.4	80.50±3.3*	108.60±4.7*	158.40±5.6*	240.20±23.9*	243.20±20.1*	245.32±22.5*
500 mg/kg/day	40.12±1.1**	58.38±2.1**	66.20±0.4**	69.43±4.4**	95.24±2.0**	148.68±8.9**	213.63±16.6**	222.63±17.3**	232.34±18.4**

*: Significant difference between control and treatment groups.

* p<0.05,

** p<0.01

**Table II:** Comparison of mean (±SEM) sperm density (million/ ml) and percent of sperm with normal morphology (%) in control and DEHP-treated Wistar rats’ offspring during different stages of postnatal development

**Groups**	**Days ** **after birth**	**Sperm count (million/ml)**	**Normal morphology (%)**
Control			
	60	65.17±2.47	82.40±4.17
	90	66.31±3.11	80.09±4.46
	120	67.22±3.81	80.66±3.78
	150	68.86±4.30	81.30±3.44
10 mg/kg/day			
	60	63.20±2.14	79.94±3.45
	90	65.60±2.50	78.67±4.22
	120	66.22±3.15	79.70±3.48
	150	67.10±2.64	79.45±4.82
100 mg/kg/day			
	60	55.72±3.23*	78.31±4.67
	90	57.43±4.12*	77.27±4.62
	120	58.52±2.48*	77.61±3.73
	150	60.66±2.70*	78.18±4.25
500 mg/kg/day			
	60	47.80±2.45**	68.66±3.21*
	90	48.20±2.61**	69.51±2.68*
	120	50.53±3.81**	70.39±4.55*
	150	52.61±3.37**	72.42±3.30*

*: Significant difference between control and treatment groups.

* p<0.05,

** p<0.01

## Discussion

Present study showed that maternal exposure to Di (2-ethylhexyl) Phthalate at doses above 100 mg/kg/day during gestation and lactation affects postnatal testis development, induces histopathological effects and reduces testis weight and volume, seminiferous tubules diameter, germinal epithelium height, epididymal sperm density and percent of morphologically normal sperm in Wistar rats offspring at pre- and post- pubertal periods. 

Several studies have shown similar adverse effects caused by developmental exposure to DEHP at high doses levels above 100 mg/kg/day. Gavage administration of 750 mg/kg/day DEHP in Sprague-Dawley rats during gestational day 14 to 21 and postpartum day 1 to 3 significantly delays male reproductive system maturation, increases testicular degeneration, and reduces the weight of testis in adult males (14, 19, 29). However, dose-dependent reductions in testis weight, an increased number of testicular histological alterations and absence of spermatocytes were seen in 21 and 28 days old offspring exposed maternally to lower levels of DEHP (3.5 and 35 mg/kg/day) during gestation and lactation via the drinking water (30). 

Also, exposure to higher doses of DEHP above 14 mg/kg/day causes to histopathological changes in the epididymidis and decreases weights of several male reproductive organs (31). Furthermore, Christiansen *et al* (2010) demonstrated that DEHP exposure from gestation day 7 to postnatal day 16 at a relatively low dose of 10 mg/kg/day causes adverse anti-androgenic effects on male rat development (22). 

But in present study, no statistically significant differences were observed in sperm and testicular parameters of offspring in 10 mg/kg/day DEHP dose group. 

Our quantitative results show testis weight of offspring decreased in dose-related manner in all treatment groups, but reduced significantly only in 500 mg/kg/day DEHP dose group. In this regard, Christiansen *et al *(2010) found that testis weight reduces in offspring exposed to 100, 600, and 900 mg/kg/day via gavage from gestation day 7 to postnatal day 16 (22). Also, administration of 375 mg/kg/day DEHP to male Sprague-Dawley rats during gestational day 3 to 21 and postpartum days 1 to 21 reduces testis and anterior prostate weights in male offspring (20). 

However, gavage administration of 1000 to 2800 mg/kg/day DEHP to Sprague-Dawley rats during postpartum day 40 to 53, 60 to 73, and 105 to 114 doses not result in any reproductive or developmental alterations (32). Mylchreest *et al* (1998) showed that there are no indications of clinical signs of toxicity or significant effects on body weight or food consumption in pregnant and lactating rats at any DEHP dose levels (33). 

Kobayashi *et al* (2006) showed that prenatal and postnatal exposure to DEHP does not affect postnatal somatic growth or endocrine and physical status of male Sprague- Dawley rats (34). Also, perinatal DEHP exposure in Wistar and Sprague- Dawley rats showed reduction of testis weights in Sprague- Dawley rats, but not in Wistar rats at a dose level of 750 mg/kg/day (35). 

Wolfe and Layton (2003) mentioned that these differences may be related to use of different rat strains (31). Also, Wilson *et al* (2007) reported that in utero effects of phthalate indicates male Sprague-Dawley rats offspring display a different postnatal reproductive phenotype than Wistar male rats’ offspring (35). Furthermore, sperm density in DEHP-treated rats’ offspring decreased dose dependently and significantly in both 100 and 500 mg/kg/day DEHP doses groups at puberty and continued until 150 days after birth. 

A reduction in daily sperm production following in utero and lactational exposure to DEHP was observed in animals exposed to 15, 45, 135 and 405 mg/kg/day DEHP doses groups (36). Also, Dalsenter *et al* (2006) reported 20% reduction of daily sperm production in Wistar rats offspring exposed to 500 mg/kg/day DEHP from first day of gestation to 21 day of lactation (37). 

Present study showed that the statistically significant reductions in testicular parameters observed in DEHP- treated groups are higher at onset of puberty that spermatogenesis begin in seminiferous tubules. Furthermore, our data showed these reductions are not reversible after cessation of exposure period and remained until 150 days of age after birth. Degree of reductions of testicular parameters increased from early week to 60 days after birth, nearly by the onset of puberty, but it decreased afterward, so that it seems that testicular parameters become better gradually until 150 days after birth. 

However, present study showed that prenatal and early neonatal life are sensitive periods for induction of permanent adverse effects by DEHP on testicular parameters of the male rats offspring, so that their reproductive efficiency reduce during postpubertal period in adult male rats. It seems that prenatal exposure to Phthalate esters affect normal testis development in offspring through disruption of Sertoli cells development (38). 

Thus, exposure to Phthalate esters during the period of Sertoli cells divisions, which last from late gestation until postnatal days 14-16 in the rat (39), may result in adverse effects on the testis. Scott *et al* (2007) reported that in utero exposure to Di (n-Butyl) Phthalate reduces the abundance of Sertoli cells relative to controls (40). In this regard, it has been reported that number of Sertoli cells per testis is highly correlated with testis size and sperm production (41). 

So, any factors that affect the development of Sertoli cells likely will have important effects on spermatogenesis efficiency during adulthood. Furthermore, it has been suggested that DEHP disrupt testosterone synthesis in fetal testis, through this alter the development of tissues dependent on androgens (42). Phthalates may be acting on genes responsible for Leydig cells development and also indirectly and/ or directly on genes involved in hormone production (38).

## Conclusion

Consequently, our quantitative microscopic study showed maternal exposure to different doses of Di (2-ethylhexyl) Phthalate (DEHP) during gestation and lactation causes to permanent and dose- related reductions in sperm and testicular parameters of Wistar rats offspring during postnatal development. 

## Footnote

***This article was taken from the master’s thesis for the M.Sc. degree.***
